# HRQOL related to urinary diversion in Radical Cystectomy: a systematic review of recent literature

**DOI:** 10.1590/S1677-5538.IBJU.2018.0858

**Published:** 2019-12-17

**Authors:** Cristiano Linck Pazeto, Willy Baccaglini, Rafael Rocha Tourinho-Barbosa, Sidney Glina, Xavier Cathelineau, Rafael Sanchez-Salas

**Affiliations:** 1 Disciplina de Urologia, Faculdade de Medicina do ABC, Santo André, SP, Brasil; 2 Department of Urology, L'institute Mutualiste Montsouris, Paris, France

**Keywords:** Cystectomy, Systematic Review [Publication Type], Urinary Diversion

## Abstract

**Introduction:**

The health-related QoL is a patient-centered evaluation covering several aspects. This evaluation seems to be particularly important in patients submitted to radical cystectomy (RC) and urinary diversion with ileal conduit (IC) or a neobladder (NB).

**Objective:**

Review all recent data comparing QoL outcomes after radical cystectomy with NB and IC diversions.

**Evidence Acquisition:**

A systematic search in PubMed/Medline, Embase, and Cochrane databases was conducted according to the Preferred Reporting Items for Systematic Reviews and Meta-analyses (PRISMA) statement in December 2018. All articles published from January 01, 2012 to December 31, 2018, were included. A study was considered relevant if it compared QoL outcomes using validated questionnaires (EORTC QLQ C30, FACT-G, FACT-BL, FACT-VCI, and BCI).

**Evidence Synthesis:**

In 11 included studies, a total of 1389 participants were accounted (730 NB and 659 IC cases). The studies were conducted in 8 different countries, two were prospective, and none was randomized. There were two studies favoring results with a neobladder, 3 with incontinent diversion and 6 with no differences. The EORTC-QLQ-C30 was the most used instrument (5 studies) followed by FACT VCI and BCI (3 studies each). Given the heterogeneity of data and lack of prospective studies, a meta-analysis was not performed.

**Conclusion:**

No superiority of one urinary diversion was characterized. It seems that the choice must be individualized with an extensive preoperative orientation of the patient and their relatives. That will probably influence how the patient accepts the new condition.

## INTRODUCTION

### Rationale

The health-related QoL is a patient-centered evaluation of the impact of an illness and its consequent therapy upon a patient, as perceived by the patient. The aspects covered in HRQOL measures are physical, role and cognitive function, symptoms, global judgment of health, psychological well-being, social well-being, degrees of activities, and satisfaction with care (1). This evaluation seems to be particularly important in patients with bladder cancer submitted to radical cystectomy (RC). This surgery enrolls an ablative step with bilateral lymphadenectomy followed by urinary diversion (UD). Generally, this last step is performed using either an ileal conduit (IC) or an ileal orthotopic neobladder (NB). However, independently of the selected type, the urinary diversion has significant detrimental effects in several life aspects of individuals what makes an evaluation of the quality of life (QoL) an important consideration. Several studies have compared QoL outcomes between these two types of diversion with conflicting results. Also, a considerable number of past studies have assessed the QoL with non-validated and nonspecific questionnaires. Therefore, to date, there is not a precise data regarding the best option based on QoL outcomes.

### Objectives

This study aims to compare the two types of urinary diversion (NB and IC) in patients submitted to radical cystectomy considering the variations on HRQOL assessed by validated questionnaires in recent comparative studies.

## MATERIALS AND METHODS

### Protocol and Registration

The systematic review was reported according to the Preferred Reporting Items for Systematic Reviews and Meta-analyses (PRISMA) statement. The review was registered in the international prospective register of systematic reviews (PROSPERO, ID CRD42019139965).

### Eligibility criteria

Using the PICOS approach, we addressed the studies characteristics.

A study was considered relevant if it compared QoL outcomes between patients submitted to RC with neobladder and ileal conduit using validated questionnaires (EORTC QLQ C30, FACT-G, FACT-Bl, FACT-VCI, and BCI). The period considered was between January 01, 2012, and December 31, 2018. The following items were required: study design (prospective or retrospective), number of patients, mean age, mean follow-up (months), and type of questionnaire used. We limited our review to clinical trials and observational studies (including cohort studies). Full consideration was given too for those relevant studies that have not been published. There was no language restriction. Review articles, abstracts conferences, meta-analyses, and case reports were excluded. Studies with <40 participants were not accepted. Author affiliations were used to help identify studies for which data were reported in more than one publication.

### Data collection process

**Figure 1:**
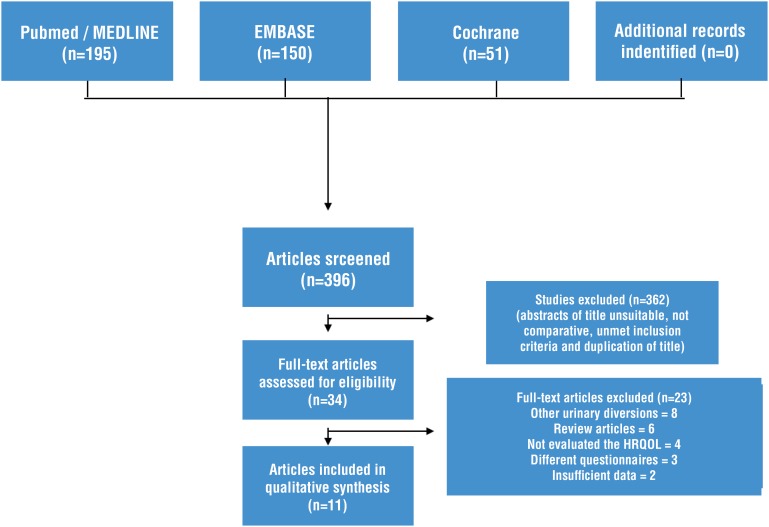
PRISMA flowchart.

A systematic search in PubMed/Medline, Embase, and Cochrane Library databases was conducted independently by two authors on December 31, 2018. The electronic search strategy (Table-1) included the following Medical Subject Headings (MeSH) terms: “quality of life” OR “QoL” AND “urinary diversion” OR “ileal conduit” OR “neobladder” AND “cystectomy.” All full-length articles published from January 01 2012 to December 31, 2018, were included in the original search. References were also examined to identify additional relevant studies (Figure-1). Missing data were requested from study authors. Discrepancies were resolved through discussion with a third author when necessary.

**Table 1:** Database search strategy.

**MEDLINE**	((((((((((“urinary diversion”) OR “ileal conduit”) OR “neobladder”)) AND ((“cystectomy”) OR “radical cystectomy”)) AND ((“quality of life”) OR “qol”)))) AND (“2012/01/01”[Date - Publication] : “2018/12/31”[Date - Publication]))		
**EMBASE**	(‘bladder cancer’/exp AND ‘cystectomy’/exp AND ‘urinary diversion/exp AND ‘quality of life’/exp) AND (2012:py OR 2013:py OR 2014:py OR 2015:py OR 2016:py OR 2017:py OR 2018:py)		
**Cochrane Library**	**ID**	**Search**	**Hits**
	#1	MeSH descriptor: [Cystectomy]	explode all trees 246
	#2	cystectomy	1351
	#3	MeSH descriptor: [Urinary Diversion]	explode all trees 314
	#4	urinary diversion	316
	#5	ileal conduit	67
	#6	neobladder	92
	#7	MeSH descriptor: [Quality of Life]	explode all trees 21532
	#8	quality of life	103764
	#9	#1 or #2	1351
	#10	#3 or #4 or #5 or #6	604
	#11	#7 or #8	103764
	#12	#9 and #10 and #11	51

### Summary measures and syntheses of results

Regardless of each study conclusions, a critical analysis was performed. Any significant difference between the mean overall/global QoL score at last follow-up was considered as favoring one of the groups regardless of specific differences in aspects of HRQOL (for example, physical or urinary functions). In those studies which a baseline QoL was not available, critical analysis and discussion between the authors were made to verify if the assumption of previous equity were possible considering pathologic and demographic data.

In the studies that used the BCI questionnaire (in which there is not precisely an overall value) differences in some of the three domains favoring any of the groups were considered.

#### HRQOL QUESTIONNAIRES EORTC-QLQ-C30

This 30-item questionnaire was designed to be cancer-specific, multidimensional in structure, appropriate for self-administration, and applicable across a range of cultural settings. This questionnaire is not a bladder cancer-specific questionnaire and information about body image, urostomy, and continence lacks. The content areas covered by the questionnaire incorporates five functional scales (physical, role, cognitive, emotional, and social), three symptom scales (fatigue, pain, nausea and vomiting), a global health status/QoL scale, and a number of single items assessing additional symptoms commonly reported by cancer patients (dyspnea, loss of appetite, insomnia, constipation and diarrhea) and perceived financial impact of the disease (2).

#### EORTC-QLQ-BLM30

The QLQ-BLM30 is a non-validated (phase III) instrument that comprises 30 questions assessing disease symptoms, side-effects of treatment, and some specific psychosocial issues of importance to patients with muscle-invasive bladder cancer. It includes questions that specifically address RC, such as urostomy problems, catheter use, and body image. The module has been developed according to the EORTC guidelines and approved after a formal review. The development process was published in the European Journal of Cancer. The QLQ-BLM-30 must be used in combination with the EORTC QLQ-C30. Modules that have completed Phase III are available for general use. However, unlike the fully validated modules, they have not undergone psychometric testing in a large international group of patients.

### FACT-VCI and FACT-Bl-Cys

The FACT-Bl-Cys (formerly the FACT-VCI) is a validated instrument specific to patients post-cystectomy. It involves a bladder cancer-specific score with 17 items about urinary (with ostomy and continence queries) bowel, self-esteem, and sexual symptoms added to the generic Functional Assessment of Cancer Therapy (FACT-G). The FACT-G is a 27-item questionnaire that measures physical, social/familial, emotional, and functional domains. The FACT-Bl-Cys total score is the sum of the four scales of FACT-G with bladder-specific subscales and range from 0-168. The higher the score, the higher the QoL. The questions BL4 (I am interested in sex) and BL5 (I am able to have and maintain an erection) are not currently scored (3, 4).

### Bladder Cancer Index-BCI

The BCI is a validated disease-specific HRQOL instrument and consists of 36 items within three principal domains (urinary, bowel, and sexual health). Each of these domains is divided into two subdomains (function and bother). The function items focus on the frequency of disease symptoms, and the bother items reflect the individual perception of these symptoms. BCI urinary items also assess daytime and night-time leakage and stoma function such as urinary leakage and skin irritation when applied in patients with ileal conduit (5).

### Critic evaluation

The Newcastle-Ottawa scale was used to access the risk of bias of individual cohort studies (Table-2). No case-control or trials were found. Given the heterogeneity of data and lack of prospective studies, a meta-analysis was not performed. To display the results, the studies were separated into three groups according to the findings.

**Table 2:** The Newcastle-Ottawa scale assessing risk of bias across the studies.

STUDIES	Anderson 2012	Cerruto 2017	Erber 2012	Gelhaus 2017	Goldberg 2016	Huang 2015	Kretschmer 2016	Large 2014	Metcalfe 2013	Singh 2014	Zahran 2017
Selection											
1. Representativeness of the exposed cohort	B	B	B	B	B	B	B	B	B	B	B
2. Selection of the non exposed cohort	A	A	A	A	A	A	A	A	A	A	A
3. Ascertainment of exposure	A	A	A	A	A	A	A	A	A	A	A
4. Demonstration that outcome of interest was not present at start of study	A	A	A	A	A	A	A	A	A	A	A
Comparability											
1. Comparability of cohorts on the basis of the design or analysis	E	ABC	E	A	ABC	D	AB	ABC	E	AD	E
Exposure											
1. Ascertainment of exposure	B	B	B	B	B	B	B	B	D	B	B
2. Was follow-up long enough for outcomes to occur	B	A	A	A	A	A	B	A	A	A	A
3. Adequacy of follow up of cohorts	A	A	A	C	C	C	C	C	D	C	D
Selection											
**Representativeness of the exposed cohort** truly representative of the average in the community somewhat representative of the average in the community selected group of users eg nurses, volunteers no description of the derivation of the cohort **Selection of the non exposed cohort** drawn from the same community as the exposed cohort drawn from a different source no description of the derivation of the non exposed cohort **Ascertainment of exposure** secure record structured interview written self report no description **Demonstration that outcome of interest was not present at start of study** yes no											
**Comparability**											
**Comparability of cohorts on the basis of the design or analysis** study controls for demographic characteristics (e.g. age, gender) study controls for any additional clinical factor (e.g. BMI, CCI, ECOG, ASA) study controls for ant additional oncology factor (e.g. TNM, local stage) study controls for time of follow-up no study control for any factor											
**Outcome**											
**Assessment of outcome** independent blind assessment record linkage self report no description **Was follow-up long enough for outcomes to occur** yes no **Adequacy of follow up of cohorts** complete follow up - all subjects accounted for subjects lost to follow-up unlikely to introduce bias - small number lost - > 80% follow up rate < 80% and no description of those lost no statement											

## RESULTS

Considering 11 included studies, a total of 1389 participants were accounted (730 orthotopic neobladders and 659 ileal conduct cases). The studies were conducted in 8 different countries: 3 in the United States, 2 in Germany, and 1 study in each following: China, Canada, Egypt, India, Italy, and Israel. None of the studies was randomized (Table-3). Half of these were published between 2015-2017. The period of studies ranged from 1991 to 2014, and the mean follow-up was 44 (range 6-121 months) and 51 (range 6-129 months) months to ileal conduit and neobladder diversions, respectively. Only two studies were prospective. The mean age of patients submitted to the ileal conduit and neobladder was approximately 68.5 years and 61.6 years, respectively. Seven studies reported a significantly higher age in IC patients compared to those of NB. Of 11 studies, 2 reported better results with neobladder, 3 with incontinent diversion and 6 no differences. Nine studies (81%) used bladder cancer disease-specific instruments. The EORTC-QLQ-C30 was the most used instrument (5 studies) followed by FACT VCI and BCI (3 studies each). The EORTC-QLQ-C30 was combined with QLQ-BLM30 module in 2 studies. Only 1 study reported performing nerve sparing (NS) procedure in all patients considered candidates for NB diversion (6).

**Table 3:** Characteristics of included studies.

							Age		Male gender		Conclusion on QoL
Study	Year	Time period	Study Type	No. (IC/NB)	Scale used	Follow-up (months)	IC	NB	IC	NB	
Anderson (4)	2012	1994-2008	retrospective	172 (71/101)	FACT-VCI	IC / NB = 12	x	x	x	x	IC better than NB
Erber(8)	2012	1993-2007	retrospective	58 (24/34)	EORTC QLQ-C30 EORTC QLQ-BLM30	IC =33.2 /NB = 50.6	median = 70	median = 62	x	x	NB better than IC
Metcalfe (10)	2013	2000-2006	retrospective	84 (53/31)	FACT-VCI	IC / NB = 67.2	mean = 68	mean = 62	x	x	No difference
Singh (9)	2014	2007-2012	prospective	164 (80/84)	EORTC QLQ-C30	IC = 24.2 /NB = 23.4	mean = 58.7	mean = 56.1	86.2%	88.1%	NB better than IC
Large (11)	2014	2011 – 2012	prospective	43 (27/16)	FACT-VCI	IC / NB = 5.8	x	x	x	x	No difference
Goldeberg (6)	2015	2004 – 2012	retrospective	95 (49/46)	BCI	IC = 46.6 /NB = 44.4	median = 72	median = 61	90%	96%	IC better than NB (urinary domain)
Huang (12)	2015	2007-2013	retrospective	117 (78/39)	BCI BIS	IC / NB = 60	mean = 64	mean = 63.6	87.1%	81.7%	No difference
Kretschmer (14)	2016	2013-2014	retrospective	100 (50/50)	EORTC QLQ-C30	IC / NB = 12	mean = 72.5	mean = 65.3	72%	74%	No difference
Cerruto (15)	2016	2010-2013	retrospective	319 (148/171)	EORTC QLQ-C30 EORTC QLQ-BLM30	IC = 44.4 /NB = 51.7	mean = 70.7	mean = 64.3	77.7%	91.2%	No difference
Gellhaus (7)	2016	1991-2009	retrospective	92 (44/48)	BCI	IC = 121.2 /NB = 129.6	mean = 67.2	mean = 58.4	80%	98%	IC better than NB (urinary function)
Zahran (13)	2017	2013-2014	retrospective	145 (61/84)	EORTC QLQ-C30 FACT-B1	IC = 60 /NB = 108	mean = 63.3	mean = 60.1	0%	0%	No difference

## SYNTHESIS OF RESULTS

### Studies Favoring Ileal Conduit Diversion

Three retrospective studies favored the IC diversion over NB. Using the FACT-VCI scores (VCI-15) Anderson et al. (4) showed that patients having an IC diversion had total scores that averaged 5 points higher than those with NB after 12 months. However, data regarding preoperative and postoperative aspects were not available. The two more recent studies evaluated the HRQOL with the BCI questionnaire. In the first one, Goldberg et al. (6) have demonstrated better urinary function scores in IC after 40 months. The urinary bother scores were similar between groups, and the prevalence of daytime and night-time leakage was higher in NB patients.

Regarding the sexual function domain, NB patients had an overall better sexual function but were also more bothered than the IC group. Those treated with NB diversion were younger and healthier. In the last study, Gellhaus et al. (7) found a better urinary function in IC patients after a mean time of 11 years (long-term HRQOL outcomes).

### Studies favoring neobladder diversion

Two studies favored the NB diversion. Using the QLQ-C30 supplemented by the BLM30 module, Erber et al. (8) have demonstrated better results in global health status/QoL and physical functioning in NB after long-term follow-up (>30 months). However, preoperative data regarding QoL lacks. Besides that, IC patients were older, presented a higher comorbidity index and more advanced diseases. Further, the BLM 30 scores (precise to evaluate urinary diversion issues) did not differ. Next, using QLQ-C30 scores, Singh et al. (9) prospectively evaluated the postoperative QoL at 6, 12, and 18 months. Continuous improvements at each time of follow-up were noted. Beyond that, NB presented higher scores in the physical, role, and social functioning and financial and global health items.

### Studies showing similar results

Six studies presented similar results between the urinary diversions. Metcalfe et al. (10) mailed questionnaires to 314 patients to access the FACT-VCI. With a response rate of 50% and a median follow-up of 5.6 years, the type of UD was not associated with QoL. Also, using the FACT-VCI, Large et al. (11) prospectively compared individuals with similar baseline HRQOL scores. After six months, a similar overall HRQOL was found. Interesting, baseline, and follow-up HRQOL scores did not differ in this last study. Huang et al. (12) compared BCI and BIS scores of two groups matched by age, gender, and ASA. Initially, NB patients have better outcomes in body image (BIS) while IC patients were better in urinary function and bother scores (BCI score). At the long-term follow-up, however, a positive effect of time was perceived in both, and the majority of differences disappeared.

Zahran et al. (13) found similar outcomes using QLQ-C30 and FACT-Bl. However, when comparing only continent NB vs. IC, better results in global health, physical, cognitive, and emotional functioning were found in NB. Also using QLQ-C30, Kretschmer et al. (14) found higher global health status as well as physical and role functioning in NB patients at three months and preoperatively. While, at 12 months, only the physical score remained higher, and the global score differences disappeared, even though a significant decrease in physical functioning occurred in both groups at 3^th^ and ^12th^ postoperative months when comparing to baseline scores. Finally, Cerruto et al. (15) performed a matched-pair analysis with comparable baseline and pathologic characteristics. NB group presented better scores in the following aspects: cognitive function, fatigue, pain, dyspnea, constipation, and flatulence. Nevertheless, no differences in global health status were observed.

## DISCUSSION

In this systematic review of HRQOL after RC, most studies were retrospective with little information about preoperative QoL. Besides, essential differences in baseline characteristics such as age, comorbidities, and pathological stage were found. Many different questionnaires were applied to evaluate the HRQOL, which gave considerably variation in the topics covered and makes comparisons very difficult. It is established that the design of the questionnaires can have profound influences on responses (1).

Some studies have reported the influence of age in HRQOL outcomes. Cerruto et al. found that patients older than 68 years presented better scores in the role, emotional, and social functioning. Also, lesser degrees of pain, financial, and future problems were described. Similarly, Goldberg et al. (6) showed a positive correlation between increasing age and urinary function score. Conversely, Metcalfe (10) described an association between younger age and increased HRQOL, while Singh (9) did not find a correlation between baseline QoL and age.

There is an intuitive thinking that continent diversion (NB) preserving the body image is associated with higher overall postoperative HRQOL. However, considering the available data, any attempt to define the superiority of one diversion from another is unfeasible. Also, the lack of randomized studies is unlikely to be supplied, considering the enormous difficulty to perform this task and ethical aspects involved. Two previous systematic reviews reported better HRQOL results in NB diversion. One carried out a meta-analysis of retrospective studies based on EORTC QLQ-C30 and presented benefits to NB over IC diversion (16). However, this study showed no difference in the majority of analysis enrolling other questionnaires (such as overall HRQOL, SF-36, and BCI forest plots). In the other review, Ghosh et al. included 22 studies-most of them reporting better results with NB (17).

All specific questionnaires in the HRQOL assessment (BCI, FACT-Bl, and BLM30) include questions related to sexual activity. However, it does not make sense, at least in men, to compare these results when no nerve-sparing procedures were pursued-the only study that did so reported performing exclusively for NB candidates (6).

Significant scores differences can be noticed through the studies. In Singh et al. (9) a progressive improvement of several EORTC QLQ-C30 domains was observed from 6 months to 18 months in NB. Thus, at 18 months, the scores were even higher than baseline levels. Differently, using the same questionnaire, Kretschmer et al. described a significant worsening in physical and role function after 6 and 12 months (14).

In most studies, the proportion of males was well above. This fact can also influence the results since the techniques differ between genders. Also, continence status following NB can be worse among women (18). Cerruto et al. (15) described that male gender was independently associated with better HRQOL scores in physical functioning, nausea, appetite loss, and future perspective. Conversely, Large et al. (11) described that HRQOL was not associated with gender in a multivariate analysis.

The ultimate success in NB diversion is strongly correlated with the continence status. Even so, a significant number of studies still pooled pad-free and 1-pad/day cases when assessing this issue. Also, more precise information about pad size and wetness is rarely reported. The number of RC by each center should be considered too since better continence rates were described in high volume centers. According to Zahran et al. (13), the nocturnal incontinence (NI) was one of the most bothersome complications in NB. These authors have shown that NB women with NI presented significant lower scores than IC women in all EORTC-QLQ-C30 domains. Moreover, higher ICIQ-SF scores were independent predictors for decreased global health scores (QLQ-C30) (14). This strong association between continence and QoL reinforces the importance of nerve-sparing during RC.

In most of the studies, an evaluation of preoperative HRQOL was not performed. This information is essential since the postoperative HRQOL levels are closely related to those at baseline (19). Similarly, the criteria to decide which type of urinary diversion were not clear. Thus, the patient's participation in the UD selection seemed very infrequent and passive. It might be that the urologist's preferences mainly drove the urinary diversion selection rather than the patient's needs and anxieties (e.g., performing the UD that the surgeon is most comfortable doing).

It is essential to discuss with the patients and families the selection of UD considering lifestyle and individual preferences. That will probably influence how they adapt and accepts the new condition. If accurate preoperative expectations are set, patients may be more content with their postoperative condition. In some cases, those things that most concern the patients are not the UD itself but the overall impact that treatment will have in the relationships and financial domains, for example. Thus, considering that either IC or NB have been reported equivalent clinical outcomes, a patient-centered decision is crucial. Patients present varied expectations in physical, health, and social domains. Recently, it has been proposed scales to measure concordance of patient's goals and UD surgical outcomes. Alignment or dissonance (also called cognitive dissonance) between patient expectation and UD outcomes may be translated into scales providing additional information to guide UD choice (20).

Generally, both urinary diversions are well tolerated. It seems that IC patients might cope with the new situation better than expected and therefore diminishing pre and postoperative differences in QoL. The similar bother scores in urinary, and bowel domains reinforce that impression (6). Kulaksizoglu et al. (21) described that HRQOL measures stabilize after 12 months, suggesting that this is the time required for adaptation. This phenomenon can partly explain the lack of difference in postoperative HRQOL in some studies. Another explanation could be the relatively imprecise data concerning continence status (21). Even so, the outcomes are still surprising since it was expected that a more complex and technical demanding diversion would reach higher HRQOL scores. It also helps to understand the worldwide decline trend in the use of NB after 2008 (22, 23).

Finally, it is essential to highlight that some studies applied the EORTC-QLQ-C30 questionnaire alone (9, 14). Although this is a validated tool for HRQOL assessment, it does not include specific questions about urinary function and incontinence.

Among the limitations of this study, we highlight the absence of high-level evidence and well-designed trials addressing this topic. The existence of several HRQOL questionnaires, scarcity of baseline scores, and continence status, and finally, the important heterogeneity between the results also contributes to the difficulty.

## CONCLUSIONS

After performing this systematic review of the recent literature, it was not possible to characterize the superiority of one urinary diversion over another in terms of HRQOL. Due to the significant heterogeneity of the studies with different questionnaires, follow-up time, and patient profiles, it is challenging to compare the available evidence. It seems that the choice of the UD must be individualized with an extensive preoperative orientation of the patient and their relatives. That will probably influence how the patient accepts the new condition.
